# Long-term effects of a severe tropical cyclone on coral reef habitat and fish assemblages at the Whitsunday Islands, central Great Barrier Reef

**DOI:** 10.1371/journal.pone.0329995

**Published:** 2026-02-25

**Authors:** Maya Srinivasan, Gemma F. Galbraith, Daniela M. Ceccarelli, Benjamin J. Cresswell, Sina J. Strähl, David H. Williamson

**Affiliations:** 1 Centre for Tropical Water and Aquatic Ecosystem Research (TropWATER), James Cook University, Townsville, Queensland, Australia; 2 College of Science and Engineering, James Cook University, Townsville, Queensland, Australia; 3 Australian Institute of Marine Science, Townsville, Queensland, Australia; 4 Great Barrier Reef Marine Park Authority, Townsville, Queensland, Australia; Secretariat of the Pacific Community, NEW CALEDONIA

## Abstract

Coral reef habitats and associated fish communities can be severely impacted by physical disturbances such as storms and cyclones, which can dramatically reduce live coral cover. However, rapid coral recolonisation and growth can lead to short-term recovery of both coral and fish assemblages. Here we examine the impact of a category 4 cyclone at the Whitsunday Islands (Cyclone Debbie in 2017). Changes in hard coral cover and the density, species richness and species composition of butterflyfishes and damselfishes were assessed over a 12-year period: 3 surveys prior to the cyclone (2012, 2014 and 2016), two surveys 8–19 months after (2017 and 2018) and another two surveys 5–6 years later (2022 and 2023). The percent cover of complex corals and massive/encrusting corals declined by 69% and 37% respectively between the pre-cyclone period and the two 8–19 months after the cyclone, with no significant recovery 5–6 years later. Density and species richness of both butterflyfishes and damselfishes declined significantly immediately after the cyclone, and these declines continued 5–6 years on. There was a shift in species composition in both fish families from a dominance of coral dependent species prior to the cyclone towards a dominance of macroalgal and rubble associated species. Species-level patterns were examined for the most abundant butterflyfish and damselfish species surveyed, and all but one species suffered long-term declines in density ranging from 31% to 85% over the period 2016–2023. The immediate and long-term declines included fishes both reliant and not reliant on live coral for habitat/food. Our study indicates that severe tropical cyclones can lead to short- and long-term declines in coral and fish assemblages, and rapid recovery should not be assumed. Given the potential for an increase in the frequency of severe cyclones with climate change, continued long-term monitoring is essential to examine the cumulative decadal effects of these disturbances.

## Introduction

Coral reefs are among the most diverse ecosystems on earth, and have immense aesthetic, cultural and economic value to humans. In recent decades, they have been facing widespread degradation due to a range of disturbances such as tropical storms, flooding, sedimentation, coral bleaching, overharvesting and crown-of-thorns seastar outbreaks, all of which are damaging to corals and the fishes that are associated with them [1–6]. Although reefs can be resilient, capable of either resisting major change or recovering from disturbance events [3,7,8], the cumulative effect of multiple disturbances that are increasing in frequency and intensity is a long-term decline in coral cover worldwide [3,4].

Tropical cyclones, hurricanes and storms can have a devastating impact on coral reef habitats and the fish communities they support [4,8–11]. The wave action caused by a tropical cyclone or hurricane can cause severe damage to coral reefs, dramatically reducing coral cover and structural complexity [2,9,10,12,13]. The physical destruction is often followed by heavy rainfall, which causes increased sedimentation and turbidity, as well as reduced salinity and temperature [4,11,14]. To a large extent, severe tropical storms can be viewed as a natural disturbance, with rapid decline and recovery a normal part of coral reef dynamics and playing a role in maintaining coral reef biodiversity [15–17]. There are many examples of coral reefs and associated fish assemblages showing substantial and sometimes complete recovery a few years after severe storm damage [8,18,19]. However, cyclones and hurricanes are now thought to have a partially anthropogenic component, in that the frequency of severe cyclones and hurricanes has been predicted to increase due to climate change [20,21]. On the Great Barrier Reef, three category 5 cyclones occurring over a 5-year period (2009–2014) caused widespread coral damage and corresponding declines in the abundance and species richness of fish communities, with little time for recovery between these events [22]. There is increasing evidence that severe cyclonic events can lead to shifts in benthic community structure that can persist for 5–10 years [5,13,22,23].

The severity of the impact of a disturbance can differ among reefs, or among different parts of a reef, depending on the level of exposure to waves and currents, and the condition and species composition of the pre-disturbance coral assemblage [2,9,13,23]. The windward sides of reefs commonly experience more severe damage from storms and cyclones than the sheltered sides [8,11,18,24,25]. The morphology of a coral species can also determine its susceptibility to disturbance [26]. Branching, digitate and plate corals (e.g., *Acropora spp.*) are typically fast growing [27] but due to their structural complexity they are highly susceptible to damage caused by increased wave action [2,13,28], while massive and encrusting coral forms are slow growing but are more robust to physical disturbances [2,13]. Because the impact of a cyclone or severe storm can vary depending on the species composition of the coral community [9,11,27], assessments of the effects of a cyclone should include data documenting the reef’s assemblage structure prior to the cyclone.

A large proportion of coral reef fish species can be classified as coral-associated due to their dependence on coral for food, shelter, reproduction, and/or for larval settlement habitat [6,7,29]. The degree of specialisation on live coral varies among fish species, and coral specialists are the most susceptible to coral loss [6,30]. Declines in their preferred habitat typically cause increased competition and susceptibility to predation, which can result in reduced abundance and possible local extinctions [3,7]. Therefore, changes in coral cover and species richness can affect fish community structure [1, 6, 8, 22, 30-32]. Severe storms that reduce the 3-dimensional complexity of a reef are likely to affect fish abundance and species richness in 3 ways: coral mortality having a negative impact on corallivores and coral dwelling species; reduced complexity having a negative impact on other species; and the sheer force of the cyclone waves having a direct impact on fish as a result of mortality, injury or stress.

The butterflyfishes (family Chaetodontidae) and damselfishes (family Pomacentridae) have numerous species known to be highly dependent on live coral for either shelter, food, or both. Butterflyfishes account for approximately half of the corallivorous fish species of the world [33] with the level of specialisation varying among species [34]. The damselfish have a large proportion of species which inhabit live coral, but there are species which thrive in degraded, algal dominated areas [35], therefore coral decline can lead to a shift in fish species composition to a higher proportion of algal associated fish species. Effects of dramatic habitat change on butterflyfishes and damselfishes are often immediate, but there can also be time lags in the effect of coral loss that may take years to observe [4,34]. For example, adults of some butterflyfish species might be able to withstand a disturbance event, but recruitment may subsequently fail due to a lack of suitable habitat and food for newly settled larvae, and a decline in abundance is only visible in the next generation [34]. Given that many reef fish species are relatively long lived, i.e., 10 years or more [35], assessing the impacts of cyclones requires long-term monitoring to capture their ecological consequences.

In late March 2017, Severe Tropical Cyclone (STC) Debbie, a slow-moving category 4 cyclone with winds of up to 250 km/h and wave heights of at least 8 m moved through the Whitsunday Island region of the Great Barrier Reef (Bureau of Meteorology 2018, Fig 1). The cyclone remained almost stationary for approximately 18 hours off the northern end of the Whitsunday Islands [36]. This study used data from a long-term monitoring program on inshore island fringing reefs in the Great Barrier Reef Marine Park to examine the impacts of STC Debbie and addressed the following questions: (1) What was the impact of STC Debbie on the percent cover of corals with complex morphologies (e.g., branching, foliose and plating) and corals with encrusting or massive morphologies, and was there any recovery in the 5 years after the cyclone?; (2) How did the abundance and species richness of butterflyfishes and damselfishes change after the cyclone, and was there any recovery or longer time lags in decline for species in the two fish families 5 years after the cyclone?; and (3) Were changes in fish community structure and the abundance of common species related to changes in habitat structure?

**Fig 1:**
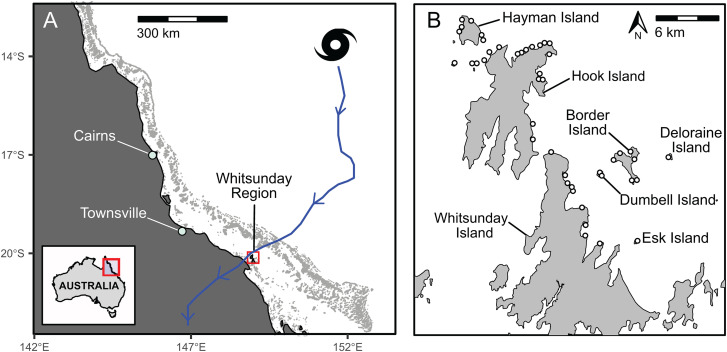
Whitsunday Islands, track of STC Debbie, and survey sites. Maps showing the location of the Whitsunday Islands on the east coast of Australia and the track of STC Debbie **(A)** and the 43 survey sites around the Whitsunday Islands surveyed between 2012–2023 **(B)**. Maps were generated in R using the ozmaps and sf packages, which use publicly available geospatial data from Australian government sources.

## Materials and methods

### Study location and survey sites

This study was conducted on fringing coral reefs around the Whitsunday Island group, within the central region of the Great Barrier Reef Marine Park from 2012 to 2023. In March 2017, the island group was in the direct path of STC Debbie, a slow-moving category 4 cyclone (Fig 1a) and was subject to destructive winds of up to 250 km/h. Benthic and reef fish communities have been surveyed at 43 sites in the Whitsunday Island group (Fig 1b) every 1–3 years from 1999 to 2023. Coral cover was very similar between survey periods from 1999 to 2016, despite a category 3 cyclone (STC Ului) passing over the Whitsundays in March 2010, therefore we used just 3 surveys prior to STC Debbie (October-November 2012, September 2014, September-October 2016) to represent baseline conditions. Four surveys have been conducted since STC Debbie, first in November-December 2017, eight months after the cyclone, then in October of 2018, 2022 and 2023. It was not possible to conduct surveys in the months immediately after the cyclone as buildings and infrastructure in the region were severely damaged, and sediment remained suspended in the water column for 3–4 months, rendering underwater visibility inadequate for reliable fish surveys.

Survey sites on the east, south, and northeast sides of islands were classified as exposed (18 sites) or semi-exposed (4 sites), and those on the west and north sides of islands were classified as sheltered (21 sites) based on the prevailing south-easterly trade winds. However, this exposure classification does not reflect exposure during a cyclone, as wind direction changes substantially as the eye passes, and different sides of islands may be exposed at different times.

### Fish and benthic underwater visual census

At each site, underwater visual surveys along five replicate 50m transects were conducted on SCUBA by three divers approximately halfway down the reef slope (typical depth 6-8m). For each transect, two divers swam side-by-side counting the larger, non-cryptic mobile fish species (which included the butterflyfishes) in a 6 m wide band, with the third diver following behind laying out the transect tape. On the return pass one diver then counted the smaller non-cryptic fish species (which included the damselfishes) in a 2 m wide band, whilst another diver conducted benthic surveys, and the third diver rolled up the transect tape. This method minimises the effect of diver avoidance by the larger, more mobile fish species which can affect abundance estimates of some species. Prior to 2022, some damselfishes (*Abudefduf* spp) were recorded to genus level and three species (*Neopomacentrus azysron*, *N. bankieri* and *N. cyanomus*) were not recorded due to extreme patchiness. These species were omitted from density and species richness analyses. Fish surveys were approved by James Cook University’s Animal Ethics Committee under approval number 22A-2840, and were carried out under Marine Parks permits G22/46858 and G22/47652.1 issued by the Great Barrier Reef Marine Park Authority.

Percent cover of benthic organisms was estimated using the point intercept method, where the benthic organism or substrate directly underneath points along the transect tape was identified and recorded. Surveys conducted before 2022 involved recording what was under each metre mark along the transect tape, i.e., 50 points per transect, and data were standardised to percent cover by multiplying by two. Surveys conducted in 2022 and 2023 used 100 points marked at random intervals along the transect tape. Hard corals were classified as live (including bleached) or dead and placed into morphological categories (branching, digitate, plate, foliose and massive). Corals in the genus *Acropora* were recorded separately and placed into morphological categories (branching, digitate and plate). Other benthic categories recorded were soft coral, sponges, macroalgae, turf algae on dead coral/limestone, coral rubble, terrigenous rock, and crustose coralline algae.

### Statistical analysis

All statistical analyses were conducted in R 4.14 [37] and the packages “tidyverse” [38], “ggplot2” [39], and “cowplot” [40] used for data wrangling and visualisation. Live hard corals were grouped into two broad categories: 1) live complex coral which included *Acropora* corals of all morphologies, and all other branching, foliose and digitate coral; and 2) live coral with massive and encrusting morphologies. The two reef fish families Chaetodontidae and Pomacentridae were selected for analysis as these two families include the most species that are highly reliant on live coral and are therefore expected to be the most susceptible to reductions in coral cover. Total species richness (S) and density (number of individuals) were calculated using the package “vegan” [41] for each family. As Chaetodontids were recorded in a 6 x 50 m belt (i.e., 300m^2^ area) on the first pass of each transect, counts were standardised to number of individuals per 100m^2^, to be consistent with the Pomacentrids recorded on the return 2 x 50 m belt transect. Total species richness for each family was the total number of species recorded in all 5 transects at each site.

To examine differences in coral cover and fish richness and density before, 8–19 months after and > 5 years after the impact of Cyclone Debbie, survey years were grouped into three time periods: Pre-cyclone (2012, 2014 and 2016), Post-cyclone 1 (2017 and 2018) and Post-cyclone 2 (2022 and 2023). We fitted generalised linear mixed effects models (GLMMs) using the package glmmTMB [42] for each response variable with time period (Pre-cyclone, Post-cyclone 1 and Post-cyclone 2) as a fixed factor and site as a random factor. Each response variable was modelled using a suitable error distribution and corresponding link function, and model fit was inspected in the DHARMa package using standard model residual diagnostics [43]. GLMMs for complex and massive/encrusting coral were fit with a negative binomial distribution log link and zero-inflation term. The Chaetodontidae species richness and density GLMMs were both fit with a Poisson distribution, log link and zero-inflation term. The GLMM for Pomacentridae species richness was fit with a gaussian distribution and identity link and the GLMM for Pomacentridae density fit with a negative binomial distribution and log link. Estimated marginal means and pairwise contrasts between time periods were calculated for each GLMM in the package “emmeans” [44].

Distance-based redundancy analysis (dbRDA) [45] was used to examine habitat-related drivers of change in multivariate community structure for *Chaetodontidae* and *Pomacentridae* communities amongst the three time periods (Pre-cyclone, Post-cyclone 1 and Post-cyclone 2). db-RDA was implemented for each family using the package vegan [41] with complex corals, massive/encrusting corals, rubble, turf algae and macroalgae fitted as habitat variables and fish community as a Bray-Curtis dissimilarity response matrix. Other benthic categories such as soft corals, sponges and terrigenous rock had much lower percent cover and were excluded from these analyses. Wisconsin double-standardisation was used to reduce the influence of numerically dominant species. A permutational ANOVA-like procedure was used with 9999 permutations to test the overall significance of the model, the significance of the environmental predictors and the significance of each axis [46]. An adjusted R^2^ (Adj. R^2^) was obtained using the package MuMIN [47] to measure the strength of the relationship between the fish community response matrix and the explanatory environmental matrix [48]. We also conducted a permutational multivariate analysis of dispersion (PERMDISP) using the function betadisper to test for Homogeneity of dispersion among the three time periods.

Finally, the 10 damselfish species and the 3 butterflyfish species with the greatest average densities across all transects and time periods were selected to examine species-level trends. These species all had mean densities of at least 1 individual per 100m^2^ during the 3 years prior to STC Debbie. For each species, individual GLMMs were fitted using glmmTMB [42] for each species. For each model, fish density was fitted as the response variable, survey year as a fixed effect and site as a random effect. Model, fit and model assumptions were examined using the DHARMA package [43]. Models for all species were fit with a negative binomial error distribution and log link. Pairwise comparisons of mean density between years were calculated as contrast ratios using the package emmeans [44].

## Results

### Effects of STC Debbie on coral cover

**Fig 2:**
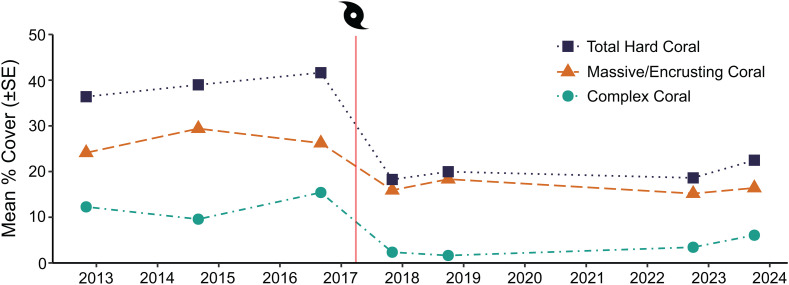
Coral cover (2012–2023). Mean percent cover (±SE) of all hard corals, massive and encrusting corals and complex corals in the Whitsunday Islands. Tick marks indicate the beginning of each year, and the placement of points between tick marks indicate the time of year of the survey. The vertical red line and cyclone symbol represents the timing of STC Debbie on 25^th^ March 2017. Standard error was calculated for each year but error bars are not visible due to very low values (<1%).

**Fig 3:**
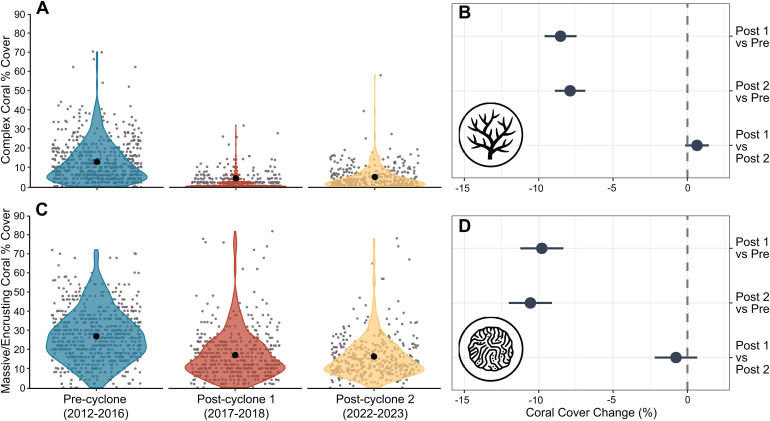
GLMM model outputs comparing coral cover among 3 time periods. Model output for GLMMs testing differences in percent cover among three time periods for complex corals **(A)** and massive and encrusting corals **(B)**. Estimated marginal means and 95% confidence intervals are plotted for each time period, with raw data as grey points and data distribution as coloured violin plots. 95% CIs are too small to be visualised. Pairwise comparisons with 95% confidence intervals are presented as contrast estimates of coral cover change between time periods. The grey dashed line in each contrast panel represents an interpretation of the contrast significance where this difference is considered significant if the confidence interval does not cross zero. Time periods are: Pre-cyclone (the 3 years prior to STC Debbie), Post-cyclone 1 (2017 and 2018) and Post-cyclone 2 (2022 and 2023).

Total coral cover remained relatively stable and high (35–40%) between 2013 and 2016, but then declined by over 50% when STC Debbie struck between the 2016 and 2017 surveys (Fig 2). There was a significant difference between the Pre-cyclone period (2012, 2014 and 2016) and the Post-cyclone 1 period (2017 and 2018, 8–20 months after the cyclone respectively) in the mean percent cover of both complex and massive/encrusting corals; however, complex corals, already less abundant before the cyclone, suffered the greatest decline (Figs 2 and 3).

There was no evidence of recovery of the coral habitat for the duration of the study period. Although there was an increase (17%) in complex coral cover between the two Post-cyclone periods (Fig 3A), this was not statistically significant (Fig 3A). There was also no significant difference in the cover of massive and encrusting corals between the two Post-cyclone periods (Fig 3B). Overall, there was a 64% reduction in complex coral cover and a 40% reduction in massive and encrusting coral cover between the Pre-cyclone and Post-cyclone 2 periods.

The majority of survey sites were severely impacted by STC Debbie, including sites that were considered sheltered under prevailing southeast trade wind conditions. Just 7 of the 43 sites experienced minimal damage, including 2 sites on the northwest side of Hook Island and 2 sites at Black Island (a small island to the west of Hook Island), which have islands all around them. The remaining relatively undamaged were sites were at Border Island, one in a sheltered bay on the north side, and two on the west side, which may have gained some shelter from Whitsunday Island to the west.

### Effects of STC Debbie on the two fish families

**Fig 4:**
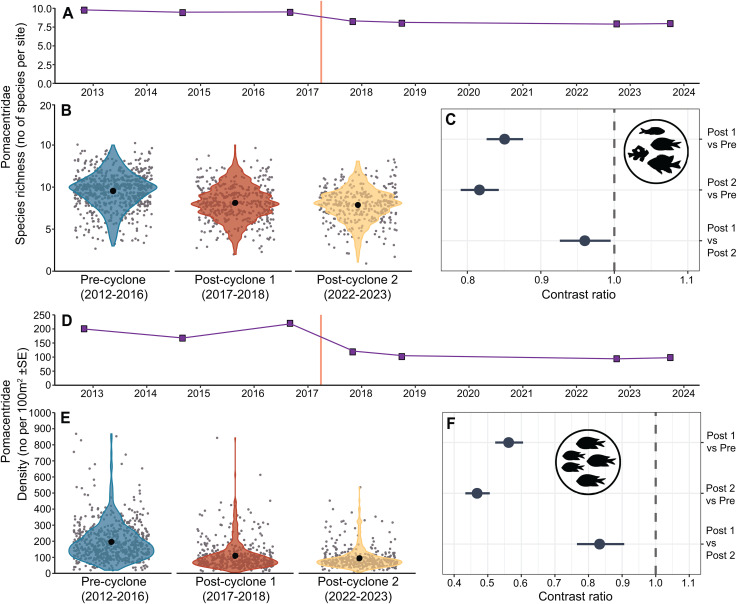
GLMM model outputs comparing damselfish species richness and density among 3 time periods. Model output for GLMMs testing differences in damselfish species richness (A-C) and density (D-F) among three time periods. Estimated marginal means and 95% confidence intervals are plotted for each time period **(A and D)**, with raw data as grey points and data distribution as coloured violin plots **(B and E)**. 95% CIs are too small to be visualised. Pairwise comparisons with 95% confidence intervals are presented as contrast ratios between time periods **(C and F)**. The grey dashed line in each contrast panel represents an interpretation of the contrast significance where this difference is considered significant if the confidence interval does not cross 1.

There was a 15% decline in damselfish species richness and a 45% decline in damselfish density from the Pre-cyclone period to the Impact period, followed by further declines of 5% in species richness and 20% in density between the two Post-cyclone periods (Fig 4A). All of these declines were statistically significant (Fig 4C and 4F). There was an overall decline of 19% in damselfish species richness and 56% in mean density between the Pre-cyclone and Post-cyclone 2 periods.

**Fig 5:**
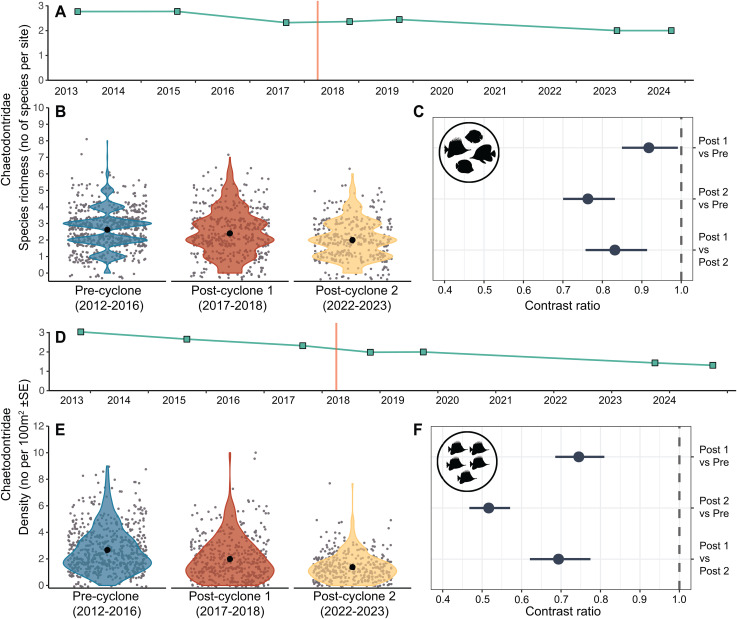
GLMM model outputs comparing butterflyfish species richness and density among 3 time periods. Model output for GLMMs testing differences in butterflyfish species richness **(A-C)** and density **(D-F)** among three time periods. Estimated marginal means and 95% confidence intervals are plotted for each time period, with raw data as grey points and data distribution as coloured violin plots. 95% CIs were too small to be visualised. Pairwise comparisons with 95% confidence intervals are presented as contrast ratios between time periods. The grey dashed line in each contrast panel represents an interpretation of the contrast significance where this difference is considered significant if the confidence interval does not cross 1.

There was a small (3%) but significant decline in mean butterflyfish species richness between the Pre-cyclone and Post-cyclone 1 periods (Fig 5A-C), and a further significant decline of 18% in butterflyfish species richness from the Post-cyclone 1 period to the Post-cyclone 2 period (Fig 5A-C). There were significant declines in butterflyfish density between the Pre-cyclone and Post-cyclone 1 periods (28%), and between the two Post-cyclone periods (31%) (Fig 5D-F). Overall, there was a 25% decline in species richness and a 50% decline in mean density between the Pre-cyclone and Post-cyclone 2 periods.

**Fig 6:**
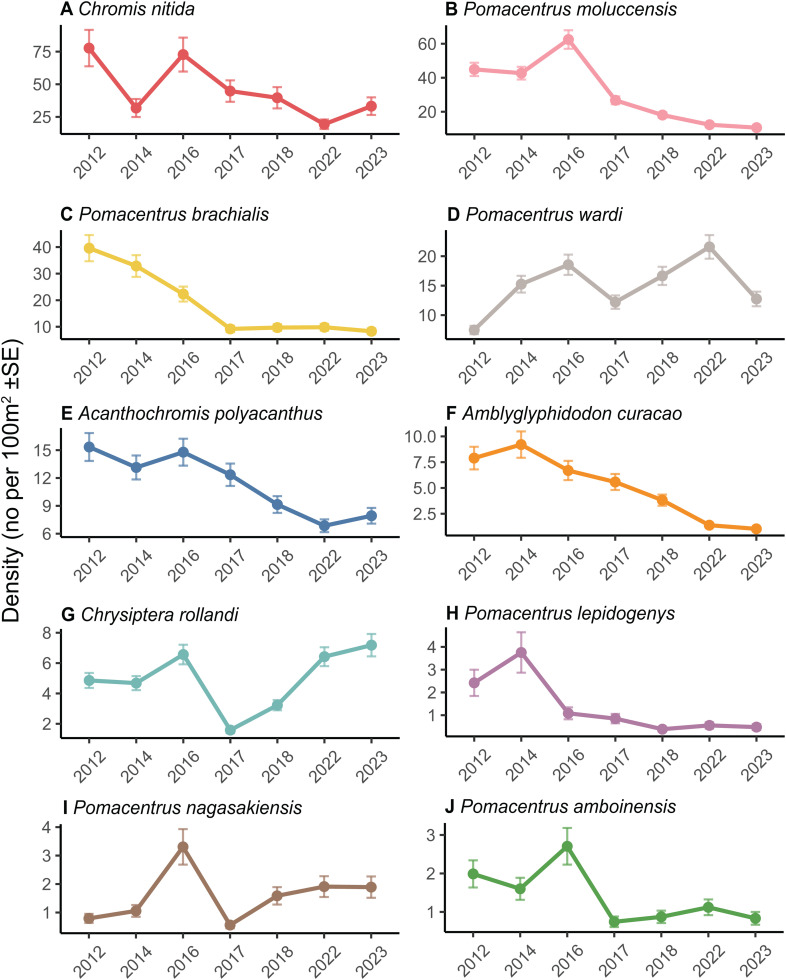
Species level patterns for the 10 most abundant damselfishes. Mean density (marginal means + /- SE) per survey year for each of the 10 most abundant damselfish species **(A-J)**. Panels for each species are arranged in order of decreasing abundance.

**Fig 7:**
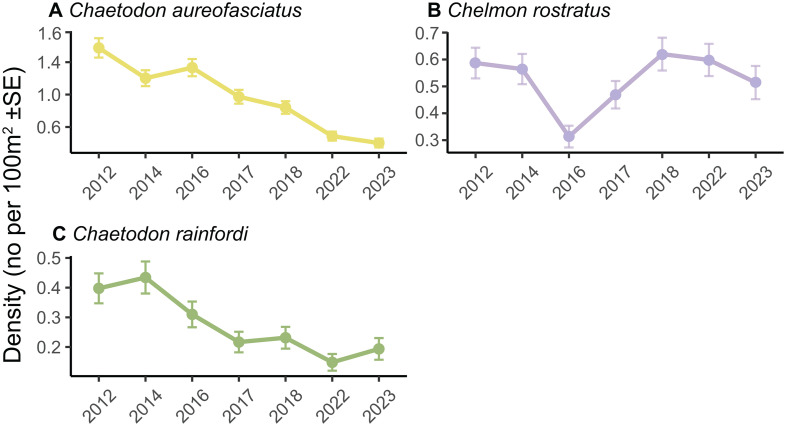
Species level patterns for the 3 most abundant butterflyfishes. Mean density (marginal means + /- SE) per survey year for each of the 3 most abundant butterflyfish species **(A-C)**. Panels for each species are arranged in order of decreasing abundance.

Species level patterns were examined for the 10 most abundant damselfish species surveyed (*Acanthochromis polyacanthus*, *Amblyglyphidodon curacao*, *Chromis nitida*, *Chrysiptera rollandi*, *Pomacentrus amboinensis*, *Pomacentrus brachialis*, *Pomacentrus lepidogenys*, *Pomacentrus nagasakiensis* and *Pomacentrus wardi*) and 3 the most abundant butterflyfish species, *Chaetodon aureofasciatus*, *Chaetodon baronessa*, and *Chaetodon rainfordi*. Ten of these 13 species had significant declines in density, ranging from 31% to 85%, in the 8-year period from 2016 (prior to STC Debbie) to 2023 (Fig 6, Table 1). *Chelmon rostratus* had an overall increase in density of 65% between 2016−23, while another species, *Chaetodon aureofasciatus*, had no significant changes in density (Fig 7, Table 1), Densities of 7 of the 13 of the species declined significantly from 2016 to 2017, with declines ranging from 27% to 83%. For two species, *P. amboinensis* and *P. brachialis*, there were no significant declines in subsequent years. *Pomacentrus moluccensis* had further significant declines in density between 2017−18 and 2018−22, and *C. aureofasciatus* had a further significant decline between 2018−22.

**Table 1:** Summary of pairwise contrasts between years. Pairwise contrasts between pairs of years from 2016 onwards, and between 2016 and 2023 (i.e., the overall change between the survey before STC Debbie and the most recent survey) for the 10 most abundant damselfish species and Chaetodon aureofasciatus. For significant contrasts, the percent change from one year to the next is given, with + or – indicating increases or declines in density. Non-significant contrasts are indicated by NS.

Species	2016 vs 2017	2017 vs 2018	2018 vs 2022	2022 vs 2023	2016 vs 2023
*Acanthochromis polyacanthus*	NS	−26%	−25%	−16%	− 46%
*Amblyglyphidodon curacao*	NS	−32%	−64%	−25%	− 85%
*Chromis nitida*	NS	NS	−51%	NS	− 54%
*Chrysiptera rollandi*	−76%	+103%	+99%	NS	NS
*Pomacentrus amboinensis*	−73%	NS	NS	NS	− 69%
*Pomacentrus brachialis*	−59%	NS	NS	NS	− 63%
*Pomacentrus lepidogenys*	NS	−54%	NS	NS	− 55%
*Pomacentrus moluccensis*	−57%	−33%	−32%	NS	− 83%
*Pomacentrus nagasakiensis*	−83%	+184%	NS	NS	− 42%
*Pomacentrus wardi*	−34%	+37%	+30%	−41%	− 31%
*Chaetodon aureofasciatus*	−27%	NS	−42%	NS	− 70%
*Chaetodon rainfordi*	NS	NS	NS	NS	NS
*Chelmon rostratus*	NS	NS	NS	NS	+65%

For two species, *A. polyacanthus* and *A. curacao*, density did not change significantly between 2016 and 2017, but then there were significant declines ranging from 16% to 64% between 2017−18, 2018−23, and 2023−24 (Fig 6, Table 1). The density of *Chromis nitida* declined by 51% between 2018−22, but the other post-cyclone pairwise contrasts were not significant. *Pomacentrus lepidogenys* had lower density in 2016 compared to 2012 and 2014, and the only pairwise contrast that was significant following STC Debbie was the one between 2017−18.

*Chrysiptera rollandi* was the only focal species to decline and then fully recover its pre-cyclone abundance. It had a significant decline (76%) in density from 2016 to 2017, but its density then doubled from 2017 to 2018, and doubled again from 2018 to 2023 to reach its pre-cyclone density. The density of *P. nagasakiensis* declined (34%) from 2016 to 2017, and then increased between 2017−18 by over 3 times, with no further change in density. The density of this species had increased between 2014−16, so although there was an overall decline of 42% between 2016 and 2023, its density in 2023 was higher than in two of the 3 years prior to the cyclone, i.e., 2012 and 2014. *Pomacentrus wardi* density declined by 34% between 2016−17, increased between 2017−18 (37% increase), and 2018−23 (30% increase), but then declined by 41% from 2022 to 2023, with an overall decline of 31% over the period 2016−23 (Fig 6, Table 1).

### Linkages between fish assemblages and changes in habitat

Distance-based redundancy analysis revealed that there were clear shifts in species composition in both fish families across the three time periods, each with significant PERMANOVA results (Damselfish: F = 5.49, p < 0.001; Butterflyfish: F = 6.42, p < 0.001). The results of the PERMDISP suggested that dispersion within each time group differed significantly for both the damselfishes (F = 32.67 p = 0.001) and butterflyfishes (F = 23.6, p = 0.001). Although the dbRDAs for both damselfish and butterflyfish communities explained over 90% of the fitted variation, the total amount of variation explained by each model was relatively low (Damselfish Adjusted R^2^ 1.5% and Butterflyfish Adjusted R^2^ 1.94%).

**Fig 8:**
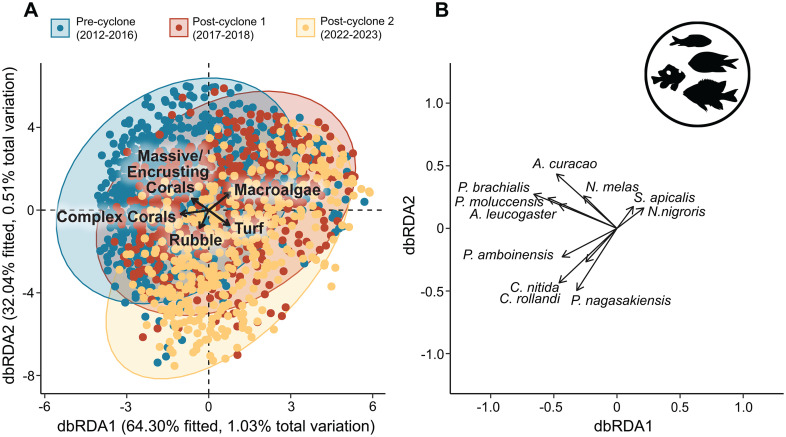
Damselfish distance-based redundancy analysis. The results of distance-based redundancy analysis (db-RDA) showing the relationship between damselfish community structure and benthic variables, and grouped by Pre-cyclone (2012−16), Post-cyclone 1 (2017−18) and Post-cyclone 2 (2022−24) time periods.

For damselfishes, the 2 major axes of ordination explained ~96.34% of fitted variation, with Pre-cyclone and Post-cyclone 1 communities separating from Post-cyclone 2 communities along dbRDA axis 2 (Fig 8). Each of the habitat terms in the damselfish dbRDA were significant (Complex coral cover, F = 9.52, p = 0.001; Massive/encrusting coral cover, F = 5.90, p = 0.001; Rubble, F = 2.87, p = 0.001, Turf algae, F = 3.51, p = 0.001 and Macroalage, F = 5.63, p = 0.001) with Pre-cyclone communities correlated with higher complex coral cover along axis 1 and Post-cyclone 1 communities correlated with lower complex coral cover along the same axis. Post-cyclone 1 communities were also correlated with increasing macroalgae cover and Post-cyclone 2 communities generally correlated with lower cover of all three habitat variables (Fig 8). Individual species aligned with Pre-cyclone communities included *Pomacentrus moluccensis, P. brachialis* and *Amblygyphidodon curacao* and *A. leucogaster*. Species more closely aligned with Post-cyclone 2 communities included *Plectroglyphidodon apicalis* and *Neogyphidodon nigroris* along axis 2 and *Chrisptera rollandi, Pomacentrus nagasakiensis* and *Chromis nitida*, predominantly along axis 1.

**Fig 9:**
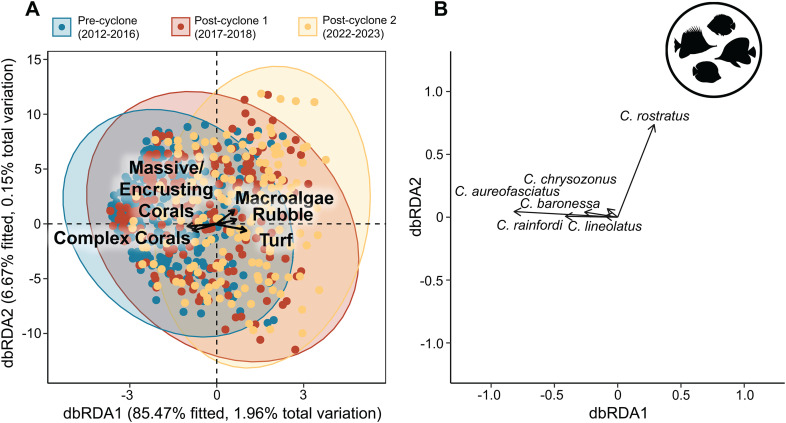
Butterflyfish distance-based redundancy analysis. The results of distance-based redundancy analysis (db-RDA) showing the relationship between butterflyfish community structure and benthic variables, and grouped by Pre-cyclone (2012−16), Post-cyclone 1 (2017−18) and Post-cyclone 2 (2022−24) time periods.

There was greater separation of community structure among the three time periods for the butterflyfishes, with the ordination of Pre-cyclone and Post-cyclone communities clearly aligned with high cover of both complex and massive/encrusting live coral cover along dbRDA axis 1 which explained 85% of fitted variation alone (Fig 9). Cumulatively the two main axes explained 92% fitted variation with most species correlated with pre-cyclone community structure along axis 1. Higher cover of macroalgae was correlated with axis 1 in the opposite direction to both complex corals and massive/encrusting corals, with *Chelmon rostratus* as the only species to show alignment with Post-cyclone 2 communities. Of the five benthic variables included in the dbRDA, complex coral cover (F = 10.7, p = 0.001), massive/encrusting coral cover (F = 13.27, p = 0.001) and turf algae cover (F = 6.31, p = 0.001) were all significant terms but not macroalage (F = 0.88, p = 0.59) or rubble (F = 0.90, p = 0.53).

## Discussion

### Overall findings

Cyclone Debbie had a devastating effect on corals in the Whitsunday Islands in 2017, particularly among corals with complex morphologies, but also among those with encrusting and massive morphologies, with very little recovery apparent 5–6 years later. Both of the two reef fish families examined declined in species richness and total density in the 8–19 months following the cyclone. In the longer-term, 5–6 years after the cyclone, there was no evidence that damselfish species richness and density were recovering, and butterflyfish species richness and density had declined further. The abundance of most of the common damselfish species and the most common butterflyfish continued to decline long after the cyclone, with reductions in density ranging from 31% to 85% between 2017 and 2024. Changes in fish communities were associated with a long-term change in habitat structure.

Our results support other studies that have reported large declines in coral cover following severe tropical cyclones [1,2,12,13] and hurricanes [5,49] with shifts in benthic community structure [2,13], and corresponding declines in reef fish diversity and abundance [1,50](1. Other studies also show no rapid recovery of corals after severe cyclones [2,5,27].

### Effects of STC Debbie on corals

The cover of all hard corals was reduced by almost 50% after Cyclone Debbie, which is a similar magnitude to other studies of severe cyclonic events. 50% declines in coral cover were reported at One Tree Island in the southern GBR following STC Hamish (category 5) in 2009 [12], and at Lizard Island in the northern GBR following STC Nathan (category 4) in 2015 [2]. In 2005, two category 4 hurricanes impacted coral reefs in Cozumel, Mexico within 3 months of each other and together caused a 56% decline in coral cover [10].

Hard corals with complex morphologies (e.g., branching, foliose and plating) had the most severe decline, with just 2% cover remaining in the two years following the cyclone, and no significant recovery apparent 5 years after the cyclone. Branching and tabulate *Acropora* species and other coral species with delicate morphologies are the most susceptible to physical damage from storms and cyclones [2,13,50], but these corals are also the fastest to recover [27]. Corals with massive and encrusting morphologies are typically more robust to physical disturbances such as storms and cyclones, but even these corals suffered a significant decline following the cyclone, to about half the cover in the pre-cyclone years. At the worst impacted sites, several massive *Porites* colonies measuring 4–6 m in diameter were observed lying upside down on the seafloor at the base of the reef with their stems broken. The lack of recovery 6 years after the cyclone indicates that it will take many years for the fringing reefs in the Whitsunday Islands to recover to their pre-cyclone levels of coral cover. A meta-analysis of 286 coral reef sites in the Caribbean found that, on average, hurricanes caused a 17% reduction in coral cover, and recovery to a pre-hurricane state took at least 8 years [5]. On the GBR, two studies documenting the recovery of corals following severe cyclones showed significant recovery in coral cover after 6–8 years, however coral cover had not returned pre-cyclone levels [12,13].

Coral recovery following a disturbance is typically through larval dispersal from other non-impacted or less affected reefs. Corals can also recover via asexual reproduction, for example the growth of fragments of branching corals, however the mortality of these fragments is very high [51]. Massive and encrusting corals can recover through the growth of remnant tissue over dead surfaces of the colony [52], however they have slow growth rates compared to fast growing branching corals, such as *Acropora*, and require extended periods of no disturbance to allow for recovery [27]. It is likely that coral recovery in the Whitsunday Islands has been slow due to the widespread nature of the impact. There are not many non-impacted areas nearby and there were very few live coral fragments that remained after the cyclone to grow and provide larvae. Additionally, there has been a shift towards algal turf and macroalgae, which has been shown to inhibit coral recruitment [53] and thus reduce the recovery potential of corals.

### Effects of STC Debbie on the two fish families

Species richness and total density of both fish families examined, the butterflyfishes and the damselfishes, declined over the 6-year period, with no recovery apparent. There have been many studies linking declines in coral cover with declines in fish abundance and species richness [1,6,54]. Declines in coral cover affect species richness and abundance through reduced habitat availability, decreased protection from predators and reduced food availability [54]. The decline in butterflyfish abundance 8–19 months after the cyclone is consistent with results from a previous study by Halford and Caley [55], which reported an immediate decline in butterflyfish density on reef slopes after a large bleaching event. However, Halford and Caley documented the beginnings of recovery 4 years after the impact. This was not the case in our study, as we observed further declines in butterflyfish density 5 years after the cyclone.

The decline in damselfish abundance in the 8–19 months after the cyclone contrasts with Halford & Caley [55], who found that damselfish density did not change immediately after a coral bleaching event but declined 5 years after the impact, i.e., there was a time lag in the effect of bleaching. Other studies have demonstrated a time lag between coral bleaching and declines in fish species richness or abundance among species able to survive without live coral, due to the time taken for the coral skeleton to break down and lose its 3-dimensional structure [4, 34, 56, 57]. In contrast, severe storms cause an immediate reduction in the 3-dimensional structure of a reef, therefore there is not likely to be a time lag in their habitat-related effects on fish, as is the case in the present study. Another reason for a time lag in the responses of fish species to coral decline is that the juveniles of many species settle on live coral but are not reliant on it as adults [4,34]. Declines in live coral would cause reduced recruitment or recruitment failure in these species, which may take several years to detect in adult populations. In this study the total densities of both families declined within 8 months of cyclone impact, suggesting impacts on adults, either directly from the force of the cyclone waves or due to the decline in coral cover. Densities continued to decline for a further 5 years, suggesting coral loss having a continued impact on adults as well as an impact on recruitment.

Twelve of the 13 species for which species level patterns were examined exhibited declines in density over at least one time period, with only one, *Chrysiptera rollandi*, recovering to pre-cyclone density over the 6-year period. *Chrysiptera rollandi* inhabits areas of rubble at a wide range of depths [58] and has previously been documented as not being strongly associated with coral loss [59]. *Chrysiptera rollandi* and two other species, *P. amboinensis* and *P. nagasakiensis*, had the highest initial declines in density (2016−17), ranging from 73 to 83%. *Pomacentrus amboinensis* and *P. nagasakiensis* are usually associated with dead corals [60] and are commonly found in patchy, rubble dominated areas at the base of the reef slope, however *P. amboinensis* juveniles typically settle into live coral [61]. None of these species rely on live coral as adults so the severity of the impact of the cyclone on their population densities suggests that the force of the cyclone waves caused direct mortality of individuals of these species, though the resuspension of sediments by cyclone waves could have also had an impact on these species or the quality of their preferred habitat. A review of the effects of storms and cyclones on coral reefs found that direct mortality of reef fishes has been reported, however it is rare and difficult to detect [11]. After the initial decline following Cyclone Debbie, the densities of *C. rollandi* and *P. nagasakiensis* increased over the subsequent years and were back to pre-cyclone levels by 2022. In contrast, *Pomacentrus amboinensis* densities remained low in subsequent years, which is most likely due to the effect of reduced coral cover on recruitment.

*Pomacentrus wardi*, a territorial farming damselfish, increased in abundance following its 2016–17 decline but then declined from 2022 to 2023, which may be natural variation, or possibly due to an increase in large, fleshy macroalgae, which has been suggested as being unfavourable to this species [59]. The remaining 6 damselfishes had overall declines in density from 2016 to 2023. *Acanthochromis polyacanthus*, *A. curacao* and *P. lepidogenys*, did not decline initially (i.e., 2016–17) but then there were significant declines in subsequent years. Although these three species are not coral specialists, the reduced structural complexity on these reefs following the cyclone is likely to have affected the survivorship of juveniles. A previous study has shown a time lag in the effects of a severe but short-lived cyclone on damselfish abundance, with no changes in abundance 6–8 weeks after the cyclone, and then declines in abundance 11–12 months after the cyclone [62]. In contrast, *P. moluccensis* density declined by over half between 2016 and 2017, which could be due to both direct mortality from the cyclone waves and reduced cover of complex corals. This species has a higher reliance on complex corals than most others examined here, inhabiting live coral as adults as well as juveniles [59].

Among the 3 most abundant butterflyfish species, one declined in density, one increased in density and one had no changes in density. *Chaetodon aureofasciatus* declined by 70% over the 7-year period which was expected as it is a corallivore and its juveniles settle into live coral [63]. However, Pratchett et al. [34] showed that a decline in live coral cover of over 90% following a coral bleaching event in the central GBR did not result in changes in the abundance of *C. aureofasciatus*, and suggested that this was due to the fact that it does also feed on non-coral prey, and prey switching following coral declines allow it to persist. The density of *Chelmon rostratus* increased by 65% between 2016−23, which is not surprising given this species is not a corallivore [64], however its density in 2016 (the year prior to the cyclone) was much lower than in the 2012 and 2014 surveys, so this increase in density may not be due to the effects of STC Debbie.

### Linkages between fish assemblages and changes in habitat

Our redundancy analyses showed that long-term changes in fish assemblages were at least partially explained by changes in habitat structure. There was a shift in species dominance among the 10 most abundant damselfish species after STC Debbie. Prior to the cyclone, the numerically dominant damselfish species were *Pomacentrus brachialis*, *Amblyglyphidodon curacao, P. moluccensis, Amblyglyphidodon leucogaster* and *Pomacentrus lepidogenys*. These species were most abundant at sites with high cover of both complex corals and massive/encrusting corals. A previous study has reported the utilization of plate corals by *P. lepidogenys* and *P. moluccensis* and utilization of branching corals by *A. curacao and A. leucogaster* [64]. *P. moluccensis* is known to aggregate in schools on *Acropora* colonies [65] and the other 3 species are also commonly associated with live branching corals [66]. Therefore, a decline in the abundance of these coral associated species with a shift in the benthic community from coral dominated to macroalgal dominated is expected.

After the cyclone, there were 2 groups of sites, one where *C. rollandi*, *P. amboinensis* and *Pomacentrus nagasakiensis* were numerically dominant and one where *P. wardi* and *Plectroglyphidodon apicalis* were numerically dominant. As mentioned earlier, *C. rollandi*, *P. amboinensis* and *P. nagasakiensis* inhabit rubble dominated areas and *P. wardi* and *P. apicalis* both inhabit patches of algal turf and macroalgae, mainly feeding on benthic algae [66–69]. Therefore, it is not surprising that there was a shift in the relative abundances of these species on the degraded reefs following the cyclone.

Among the butterflyfishes, *Chelmon rostratus* was the only butterflyfish aligned with the shift in community structure after STC Debbie. As mentioned earlier, this species is not a corallivore [64], therefore it is not surprising that its relative abundance increased with the shift in benthic community structure to higher cover of macroalgae and rubble after the cyclone, while pre-cyclone communities were dominated by corallivores such as *Chaetodon aureofasciatus* and *C. rainfordi*.

## Conclusions

Our results align with a growing number of studies showing severe responses to and slow recovery from severe cyclonic events. We documented dramatic declines in the cover of complex corals and massive/encrusting corals at the Whitsunday Islands in the 18 months after STC Debbie in 2017. Species richness and total densities of butterflyfishes and damselfishes also declined between 2016 and 2018. Coral cover did not improve between 2018 and 2023, and there were further declines in the species richness and total densities of both focal reef fish families. Six years after the cyclone, there was no recovery apparent among the corals, butterflyfishes or damselfishes, with no change in coral cover between 2018 and 2023 and further declines in the species richness and total densities of both fish families. Among the 11 fish species for which species level patterns were examined, all but one species declined in abundance over the 6-year period with declines ranging from 31% to 85%. There were shifts in the overall community structure in both fish families, with declines in coral associated species and an increase in the relative abundance of species associated with rubble and macroalgae after the cyclone.

Our fingings suggest it is likely to take many more years for these reefs to recover to pre-cyclone conditions. Given the predicted increase in the frequency of severe tropical cyclones, there is a declining probability that reefs will fully recover from these events. Continued monitoring is essential to document the longer-term outcomes of the increasing frequency of cyclones and shorter recovery periods, and what this will mean for coral reefs in the future.
